# Using *Dictyostelium* to Advance Our Understanding of the Role of Medium Chain Fatty Acids in Health and Disease

**DOI:** 10.3389/fcell.2021.722066

**Published:** 2021-09-13

**Authors:** Erwann Pain, Sonia Shinhmar, Robin S. B. Williams

**Affiliations:** Centre for Biomedical Sciences, School of Biological Sciences, Royal Holloway University of London, Egham, United Kingdom

**Keywords:** decanoic acid, diacylglycerol kinase, *Dictyostelium*, epilepsy, MCT, mTORC1, phoshoinositides, 4BCCA

## Abstract

Ketogenic diets have been utilized for many years to improve health, and as a dietary approach for the treatment of a range of diseases, where the mechanism of these low carbohydrate and high fat diets is widely considered to be through the production of metabolic products of fat breakdown, called ketones. One of these diets, the medium chain triglyceride ketogenic diet, involves high fat dietary intake in the form of medium chain fatty acids (MCFAs), decanoic and octanoic acid, and is commonly used in endurance and high intensity exercises but has also demonstrated beneficial effects in the treatment of numerous pathologies including drug resistant epilepsy, cancer, and diabetes. Recent advances, using *Dictyostelium discoideum* as a model, have controversially proposed several direct molecular mechanisms for decanoic acid in this diet, independent of ketone generation. Studies in this model have identified that decanoic acid reduces phosphoinositide turnover, diacylglycerol kinase (DGK) activity, and also inhibits the mechanistic target of rapamycin complex 1 (mTORC1). These discoveries could potentially impact the treatment of a range of disorders including epilepsy, cancer and bipolar disorder. In this review, we summarize the newly proposed mechanisms for decanoic acid, identified using *D. discoideum*, and highlight potential roles in health and disease treatment.

## Introduction

**FIGURE 1:**
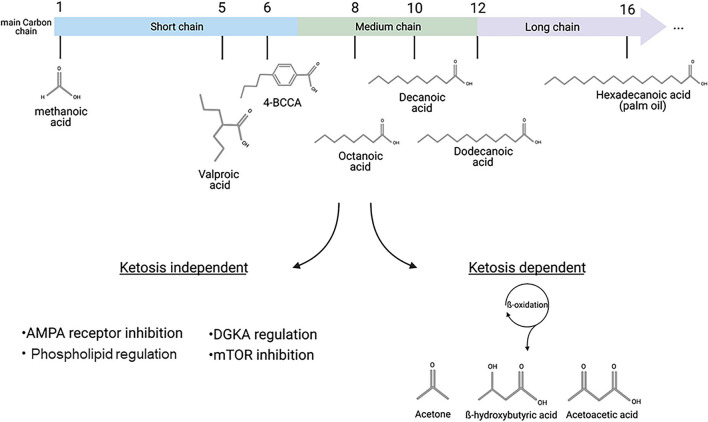
Fatty acids classification and distinct pathways associated with therapeutic mechanisms. Main carbon chain length provides the primary classification of fatty acids, including short chain fatty acid such as valproic acid, MCFAs such as octanoic or decanoic acid, and long chain fatty acids such as hexadecanoic (palmitic) acid. Medium- and long-chain fatty acids can be metabolized in a process called ketosis, where β-oxidation leads to the formation of the three ketone bodies: Acetone, β-hydroxybutyric acid and acetoacetic acid. Recent discoveries, including those based in *D. discoideum*, have identified ketosis-independent mechanisms of some MCFAs, including decanoic acid and a related trans-4-butylcyclohexanecarboxylic acid (4BCCA) acid in a range of therapeutic associated mechanisms. This figure was created with Biorender.com.

In our modern societies, fats are often considered to be damaging to health, with more than half of the European population considered overweight, and with cardiovascular diseases caused by obesity providing the most common cause of death in rich countries (Abdelaal et al., 2017). However, as primary constituents of the cell membrane, in addition to functions in the regulation of numerous signaling pathways, fats are an important group of compounds involved in normal cell physiology and health (Alves-Bezerra and Cohen, 2018; Kimura et al., 2020). Fatty acids can be classified by carbon chain length, into short chain (up to 6 carbon atoms), medium chain (7–12 carbon atoms), and long chain fatty acids (>14 carbon atoms) (Figure 1). These groups can have different physiological roles in a variety of processes including fetal development, cell metabolism, DNA regulation and inflammation (Hamosh and Salem, 1998; Tvrzicka et al., 2011; Kalish et al., 2012; Tan et al., 2014). As part of our dietary intake, fats are mainly consumed as triglycerides (three fatty acids associated with glycerol), that are broken down into fatty acids in the intestine and are then transferred to the liver where they undergo mitochondrial and peroxisomal β-oxidation (Schönfeld and Wojtczak, 2016). Under low carbohydrate conditions, the oxidation of fatty acids results in the formation of acetyl-CoA and three by-products (acetoacetate, β-hydroxybutyrate and acetone) called “ketones” or “ketone bodies,” which are circulated around the body in the blood and can cross the blood brain barrier, to be used as an energy source by neurons and astrocytes (Nguyen et al., 2008; Augustin et al., 2018a). This process of ketone body formation is increased in the absence of dietary carbohydrates in a process known as “ketosis” and is considered to offer positive effects on health and disease treatment (Wilhelm et al., 2021; Yurista et al., 2021). However, recent studies using *D. discoideum* as a model have suggested novel direct mechanisms for specific fatty acids.

Ketogenic diets were originally developed to reproduce a state of metabolic starvation (Wheless, 2008), but are currently used by healthy individuals to improve athletic performances or body composition (Paoli et al., 2015; McSwiney et al., 2018), and also in medicine for the treatment of numerous pathologies where they are collectively known as “Foods for Special Medical Purposes.” These diets are best known and validated in the treatment of epilepsy (Youngson et al., 2017; D’Andrea Meira et al., 2019), where they are currently used as an alternative to pharmacological treatments, in patients who do not respond to two or more drugs. These dietary approaches have repeatedly shown clinical efficacy in this role (Martin-McGill et al., 2018), providing better seizure control in drug resistant patients than additional pharmaceutical treatments. In addition, ketogenic diets are increasingly being investigated for the treatment of cancers (Klement, 2017), although clinical validation remains to be confirmed (Plotti et al., 2020), and in neurodegenerative disorders like Alzheimer’s disease (Augustin et al., 2018a; Rusek et al., 2019; Avgerinos et al., 2020) where they are likely to provide mild improvements in cognition.

The first widely used ketogenic diet, termed the “classical” ketogenic diet, is based upon the consumption of a very high ratio of long chain fatty acids to carbohydrates as a source of energy (90% fats to 10% carbohydrates/proteins) (Hartman and Vining, 2007), which results in ketosis. Interestingly, the mechanisms of action of the diet remain unclear (Rho and Sankar, 2008; Masino and Rho, 2012; Rho and Stafstrom, 2012) and ketones levels do not correlate with seizure control (Bough et al., 1999; Thavendiranathan et al., 2000; Dell et al., 2001; Rho et al., 2002) nor do ketones block seizure activity in various epilepsy model systems (Samoilova et al., 2010). Despite the efficacy of this diet, the high fat: carbohydrate ratio often proves difficult to maintain (Freeman et al., 2006), and numerous variations have been developed with modified fats, and differing ratios of fats to carbohydrates/proteins (Payne et al., 2011).

One variation of the classical ketogenic diet is the medium chain triglyceride (MCT) ketogenic diet that involves the dietary intake of triglycerides containing two MCFAs, decanoic (C10) and octanoic acid (C8), that are found in coconut oil. This diet has been showed to be effective in the treatment of patients with drug resistant epilepsy (Neal et al., 2009) and gives rise to ketosis, but additionally elevate levels of octanoic and decanoic acid. Studies described in this review led to the identification of potent anticonvulsant and neuro-protective effects of decanoic acid and related MCFA (Wlaź et al., 2012; Chang et al., 2013; Augustin et al., 2018a). MCFAs have since been demonstrated to have anti-cancer properties, reducing tumor size and inflammation (Narayanan et al., 2015; Wakana et al., 2019), and are also used in the treatment of metabolic disorders such as diabetes or steatohepatitis (Wang et al., 2017; Sung et al., 2018). Interestingly, several novel MCFAs related to decanoic and octanoic acid also show specific seizure control activity (Chang et al., 2013, 2014b, 2016; Augustin et al., 2018a). Thus, the mechanism of the MCT ketogenic diet in health and disease treatment may be attributed to the effects of both ketone bodies and specific MCFAs. In this review, we highlight the advantages of using *Dictyostelium discoideum* as a biomedical model and specifically outline recent discoveries made in this model leading to the demonstration of seizure control by decanoic acid and other MCFAs, and potentially in other disease treatments, as well as the translation of these studies to preclinical or human models.

### The Increasing Use of *Dictyostelium* as a Cell and Developmental Model in Biomedical Research

*D. discoideum* is increasingly being used as a model in biomedical research to further our understanding of conserved molecular pathways and cellular responses to a wide variety of pharmacological agents and natural products (Waheed et al., 2014; Paschke et al., 2018; Schaf et al., 2019). This interest is due to both a relatively simple genome, compared to animal models, with ∼12,500 genes providing much less redundancy in cellular function (Cocorocchio et al., 2018; Kelly et al., 2018; Paschke et al., 2018), and the simplicity of carrying out a range of experimental approaches that are difficult in mammalian systems. When investigating molecular mechanisms of bioactive compounds, the use of *D. discoideum* enables the production of a library of mutants lacking individual non-essential genes. This method, termed “Restriction Enzyme Mediated Integration” (REMI), involves the insertion of an antibiotic resistance gene (Blasticidin) randomly throughout the genome (Takeda et al., 2003; Kuspa, 2006). This library can be exposed to a compound of interest at a concentration that blocks wild type cell growth, enabling mutants that have lost a potential target protein to show continued growth, and thus allowing the identification of the cellular mechanism of bioactivity (Waheed et al., 2014; Gruenheit et al., 2019; Perry et al., 2020a; Damstra-Oddy et al., 2021). The use of *D. discoideum* also provides a range of useful approaches to define compound resistance, such as monitoring acute changes in cell shape or movement (Parent, 2004; Cocorocchio et al., 2018; Kelly et al., 2018), inducing autophagy (Mesquita et al., 2017), regulating cell signaling (Perry et al., 2020b; Damstra-Oddy et al., 2021) or phosphoinositide turnover (Xu et al., 2007; Chang et al., 2012). The model allows the use of plasmids to over-express fluorescently tagged proteins in isogenic culture, that define protein cellular location, restore phenotypes in mutants, and enable functional analysis of specific amino acids (Ludtmann et al., 2014). Recent development of the CRISPR-cas9 system for efficiently knocking out genes has also been demonstrated in *D. discoideum* (Sekine et al., 2018). *D. discoideum* also enables a precise and well characterized differentiation between both growth and development, where upon starvation of growing cells, transcriptional events trigger expression of a series of proteins within a defined developmental cycle (Reymond et al., 1995; Singer et al., 2019). This leads to individual cells excreting cyclic AMP (cAMP), resulting in waves of aggregation to form cell mounds, that either mature into a slug enabling phototaxis (Chisholm and Firtel, 2004; Li and Purugganan, 2011) or into a multicellular fruiting body structure (Weijer, 2004). This developmental process can also be used to define the effects of bioactive compounds in both wild type cells and in mutant cells lines lacking potential compound targets or in disease associated processes (Williams et al., 1999; Kelly et al., 2018; Fernando et al., 2020).

### *Dictyostelium* Research to Improve Our Understanding of the Cell and Developmental Mechanisms of MCFAs in Health and Disease Treatment

*D. discoideum* has played a key role in research focused on identifying and characterizing the pleiotropic effects of MCFAs. This area of research was first initiated through studies on the short, branched chain fatty acid valproic acid (VPA; 2-propylpentanoic acid), commonly used as a medicinal treatment for epilepsy and bipolar disorder (Fontana et al., 2020; Rahim et al., 2021). Surprisingly, the molecular mechanism of this drug has remained largely unclear (Terbach and Williams, 2009). Through the initial analysis of the cellular effects of VPA and other MCFAs in *D. discoideum*, there have been three significant mechanisms of MCFAs identified which have implications on disease treatment, described here.

#### Attenuating Phosphoinositide Signaling

**FIGURE 2:**
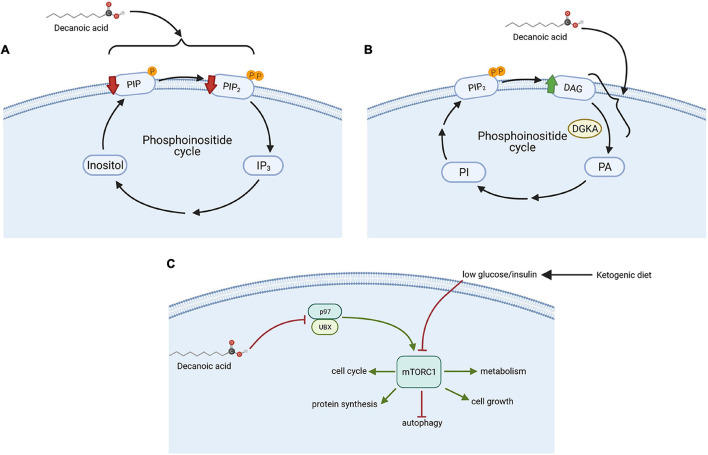
Mechanisms of action of decanoic acid identified in *D. discoideum*. **(A)** Decanoic acid regulates the phosphoinositide pathway by decreasing phosphatidylinositol phosphate (PIP) and phosphatidylinositol diphosphate (PIP_2_) concentrations; **(B)** increased diacylglycerol (DAG) levels through the inhibition of diacylglycerol kinase A (DGKA); and **(C)** reduced the activity of the mechanistic target of rapamycin complex 1 (mTORC1) independent from glucose and insulin level through the inhibition of p97, an mTORC1 activator, with a range of downstream effectors including autophagy. This figure was created with Biorender.com.

Initial studies in *D. discoideum* were the first to identify an effect of VPA in attenuating phosphoinositide signaling, through the analysis of VPA-dependent inhibition of cell movement and development in this model (Xu et al., 2007). Further studies in the model characterized the molecular mechanism for this effect (Chang et al., 2012), and ultimately focused on looking for a replacement to valproic acid that reproduces its cellular mechanism(s), leading to the identification of a range of MCFAs that show enhanced potency in regulating phosphoinositide signaling by reducing PIP and PIP_2_ levels (Figure 2A). This study provided a pivotal breakthrough in the identification of decanoic acid as reproducing the bioactivity of VPA in a more potent manner (Figure 1), that may impact on seizure control since it is a key component of the MCT ketogenic diet (Augustin et al., 2018a), and it is elevated in the plasma of patients on the MCT ketogenic diet (Huttenlocher et al., 1971; Haidukewych et al., 1982; Sills et al., 1986a, b). Other potent compounds identified in this study included the nine-carbon fatty acid, nonanoic acid (C9), and various branched chain derivatives of octanoic acid (C8). Thus, MCFAs were primarily identified to reproduce a cellular effect of VPA in reducing phosphoinositide signaling in *D. discoideum*, with potential implications on seizure control.

#### Reducing DGK Signaling

Subsequent studies employed *D. discoideum* to demonstrate that both VPA and specific MCFAs reduce the activity of diacylglycerol kinase A (DGK) (Kelly et al., 2018; Figure 2B). Mammalian (Human) diacylglycerol kinase (DGK) consists of ten isoenzymes (Mérida et al., 2008), and thus the single isoform of DGK in *D. discoideum* is likely to represent an ancestral progenitor of the various human proteins. This class of enzyme plays a key role in phosphoinositide signaling (Mérida et al., 2008), functioning by phosphorylating diacylglycerol (DAG) to form phosphatidic acid, thereby decreasing DAG levels and regulating essential lipid signaling involved in numerous functions such as membrane trafficking and cell migration (Cutrupi et al., 2000; Fang et al., 2001). Different isoforms of the mammalian DGK are involved in regulating seizure activity (Rodriguez de Turco et al., 2001; Leach et al., 2007; Ishisaka et al., 2013). Three single nucleotide polymorphisms (SNPs) in a specific isoform of DGK in humans have been linked to bipolar disorder (Baum et al., 2008; Squassina et al., 2009), and loss of other isoforms in mammalian models provides bipolar disorder-like behaviors (Kakefuda et al., 2010; Isozaki et al., 2016), including response to lithium, a mainstay in bipolar disorder treatment (McIntyre et al., 2020). In *D. discoideum*, acute VPA treatment triggers loss of cell shape, where this effect was attenuated following loss of DGKA (Kelly et al., 2018). Furthermore, chronic treatment with VPA, or lithium, or specific MCFAs including decanoic acid, blocked multicellular development, and this effect was lost in the DGKA^–^ mutant. Finally, these compounds triggered an increase in DAG levels in wild type cell, but not in DGKA^–^ cells (Kelly et al., 2018), where metabolic changes in the mutant were suggested to bypass DGKA and remove sensitivity to pharmacological inhibition of the enzyme by these compounds. Thus, this *D. discoideum-*based research suggests that VPA, lithium and specific MCFAs including decanoic acid may function in the treatment of bipolar disorder through effects on DGK.

#### Reducing mTORC1 Activity

More recently, *D. discoideum* has been employed to investigate the effect of MCFAs on the evolutionarily conserved mTORC1 complex (Warren et al., 2020; Figure 2C). mTORC1 is one of the main cellular nutrient sensors, controlling cell homeostasis by modulating cellular growth, development and autophagy (Laplante and Sabatini, 2012). In the classical ketogenic diet, mTORC1 activity is reduced through a reduction in glucose and insulin signaling (Ostendorf and Wong, 2015; Sweeney et al., 2017; Robert et al., 2018). This inhibitory activity provides a treatment approach for patients with drug-resistant epilepsies, cancers, and neurodegenerative disorders, and also may lead to lifespan extension (Laplante and Sabatini, 2012). Surprisingly, analysis of the mTORC1 complex in *D. discoideum* showed that decanoic acid (but not octanoic acid) down-regulated the activity of mTORC1 under high glucose conditions in the absence of insulin, thus conflicting with the current mechanism proposed for the classical ketogenic diet (Warren et al., 2020). To investigate this mechanism, a *D. discoideum* mutant library was screened to identify insertional mutants showing resistance to the growth inhibitory effect of decanoic acid. This approach led to the identification of a protein, UBXD18 (UBX domain protein 18) to be responsible for decanoic acid-dependent growth sensitivity, and necessary for the decanoic acid-dependent inhibition of mTORC1. These domain proteins represent very highly conserved functions in binding to the endoplasmic reticulum ATPase, p97 (Barthelme and Sauer, 2016). To identify how UBXD18 functioned in this role, both UBXD18-GFP and p97-RFP proteins were expressed together, and immune-precipitation was used to demonstrate direct interaction between these two proteins. It was also found that decanoic acid reduced p97 activity, leading to mTORC1 inhibition. Direct inhibition of p97 using specific inhibitors also reduced mTORC1 activity. Thus, the use of *D. discoideum* as a model to study the effect of decanoic acid on mTOR signaling, provided new insight to the regulation of this evolutionarily conserved complex, and uncovered a potential new target pathway for disease treatment (Warren et al., 2020).

### Validating *Dictyostelium*-Based Discoveries in Preclinical and Clinical Models

Innovative research in the fields of health and disease treatment using model systems must be validated in preclinical models to evidence potential clinical relevance. This approach has been demonstrated in *D. discoideum*-based research into short and MCFAs. The reduction of phosphoinositide levels by VPA identified in *D. discoideum* (Chang et al., 2012) was translated to *in vitro* and *in vivo* animal models, where it reduced seizure-induced depletion of a key phosphoinositide PIP_3_ in primary neurons during seizure-like activity *in vitro*, and *in vivo* in rodent brains during seizures (Chang et al., 2014a). The identification of a range of MCFAs that were more potent than VPA in *D. discoideum*, suggested that they may also provide more potent seizure control than VPA. The efficacy of these MCFAs was then validated using both *in vitro* and *in vivo* animal seizure models (Chang et al., 2013, 2014b), also providing neuroprotective effects (Chang et al., 2013), and confirming the activity of decanoic acid, 4BCCA (trans-4-butylcyclohexanecarboxylic acid), 4EOA (4-ethyloctanoic acid) and other compounds to provide strong seizure control in multiple *in vivo* seizure models (Figure 1). Finally, decanoic acid but not ketones was also shown to provide strong seizure-control effects in mammalian *in vitro* models, through the direct inhibition of α-amino-3-hydroxy-5-methyl-4-isoxazolepropionic acid (AMPA) receptors, one of the main excitatory neurotransmitter receptors in the brain, through a direct and non-competitive manner (Chang et al., 2016; Yelshanskaya et al., 2020). Specific ratios of MCFAs, with elevated levels of decanoic acid, were then defined to provide enhanced inhibition of AMPA receptors associated with seizure control, and decanoic acid provided synergistic inhibition of AMPA receptors with another epilepsy treatment targeting AMPA receptors, perampanel, in rodent and human models (Augustin et al., 2018b). Thus, in seizure control, specific MCFAs were identified and confirmed to provide potential treatments in epilepsy related disorders.

The glucose- and insulin-independent inhibition of mTORC1 by decanoic acid, identified in *D. discoideum*, was also validated in mammalian and human models (Warren et al., 2020). Initially, the effects of decanoic acid on mTORC1 activity was analyzed using a standard *ex vivo* animal model for epilepsy research, rat hippocampal slices in artificial cerebrospinal fluid. Decanoic acid treatment of these slices (1 h; 100 and 300 μM), and mTORC1 activity analysis by Western blot (Warren et al., 2020), showed that decanoic acid reduced mTORC1 activity without glucose restriction or insulin modification. This mechanism was then analyzed in an *in vitro* human model, using astrocytes from iPSCs cells derived from healthy individuals and people with tuberous sclerosis complex (TSC) mutations. These mutations give rise to elevated mTORC1 activity, and result in tumors, cognitive disabilities and epilepsy (Henske et al., 2016). This approach confirmed that decanoic acid reduced mTORC1 activity in human astrocytes, independent of glucose and insulin levels, validating the results obtained in *D. discoideum*. These translational studies confirm the effect of decanoic acid in inhibiting mTORC1 activity and are also likely to be of clinical significance in disease treatment.

The clinical relevance of decanoic acid in seizure control has recently been demonstrated. This involved the development of a new MCT blend containing elevated levels of decanoic acid, thus maximizing the therapeutic effects of the MCFA treatment. Subsequently, a recent clinical trial has employed a 12 week prospective feasibility study of this new blend, “K.Vita,” to evaluate tolerance, acceptability and compliance in children and adults diagnosed with epilepsy (ClinicalTrials.gov Identifier: NCT02825745). The diet increased plasma levels of decanoic acid by 24–28-fold (in children and adults), and provided a 50% reduction in mean frequency of seizures/events, correlating with decanoic acid levels, despite absent or very low levels of ketosis (Schoeler et al., 2021).

## Conclusion

Dietary intake of MCFAs through the MCT ketogenic diet provides a range of positive health and disease treatment effects (Neal et al., 2009; Narayanan et al., 2015; Sung et al., 2018). The current view of how this diet functions in these roles has, until recently, been solely based upon ketosis and a shift in metabolism (Wilhelm et al., 2021; Yurista et al., 2021). However, a range of experiments described in this review, initiated in *D. discoideum* and translated to preclinical models and clinical studies, has shed new light on the cellular function of the diet. These studies suggest that decanoic acid may play a direct role in seizure control, independent of ketosis, and identify several compounds (including 4BCCA and 4EOA) that are of potential interest as novel pharmaceutical approaches to replace VPA in epilepsy (and bipolar disorder) treatment. The use of *D. discoideum* as a tractable model (Schaf et al., 2019), with a low redundancy genome and a range of advantageous experimental techniques was essential to identify mechanisms and targets for MCFAs in a range of disease including epilepsy, bipolar disorder and cancer. Validation of these discoveries, through translation to mammalian or human models has confirmed these discoveries are relevant to disease treatment. Thus, *D. discoideum* provides an excellent pre-clinical model for understanding the role of current and potential new therapies, leading to clinically relevant discoveries to build a healthier society.

## Author Contributions

EP wrote the first draft and subsequent drafts of the article and constructed the figures. SS contributed to the writing. RW proposed the review, structured the sections, drafted text, and reviewed drafts. All authors contributed to the article and approved the submitted version.

## Conflict of Interest

RW has patents related to MCFAs in therapeutic use (WO2012069790A1, WO2016038379A1, WO2018189113, and WO2019002435A1). The remaining authors declare that the research was conducted in the absence of any commercial or financial relationships that could be construed as a potential conflict of interest.

## Publisher’s Note

All claims expressed in this article are solely those of the authors and do not necessarily represent those of their affiliated organizations, or those of the publisher, the editors and the reviewers. Any product that may be evaluated in this article, or claim that may be made by its manufacturer, is not guaranteed or endorsed by the publisher.
